# The independent impact of dementia in patients undergoing percutaneous coronary intervention for acute myocardial infarction

**DOI:** 10.1002/clc.23967

**Published:** 2023-01-12

**Authors:** Afek Kodesh, Tamir Bental, Hana Vaknin‐Assa, Yeela Talmor‐Barkan, Pablo Codner, Amos Levi, Ran Kornowski, Leor Perl

**Affiliations:** ^1^ Cardiovascular Department, Rabin Medical Center, Beilinson Hospital, The Faculty of Medicine Tel‐Aviv University Tel‐Aviv Israel

**Keywords:** death, dementia, major adverse cardiac event, myocardial infarction, percutaneous coronary intervention

## Abstract

**Background:**

Although age and frailty are associated with worse prognoses for patients who undergo percutaneous coronary intervention (PCI), little is known regarding the independent impact of dementia.

**Hypothesis:**

The aim of this study was to evaluate the association between dementia and outcomes for patients with acute myocardial infarction (AMI).

**Methods:**

Consecutive patients with ST‐elevation or non‐ST elevation MI who had undergone PCI as part of our AMI registry were included in this study. We compared outcomes within the 1‐year period of their PCI, including death and major adverse cardiac events (MACE) and corrected for confounders using Cox regression.

**Results:**

Of 28 274 patients, 9167 patients who had undergone PCI for AMI were included in this study, 250 with dementia; Mean age (77.4 ± 9.4 in the dementia group vs. 63.6 ± 12.7 in the control), female gender (32.4 vs. 24.2%, *p* = .003), diabetes mellitus (54.0 vs. 42.4%, *p* < .001) and chronic kidney disease (44.4 vs. 19.3%, *p* < .001) were higher. At 12 months, unadjusted rates of death (25.5 vs. 9.8%, *p* < .001) and MACE (33.8 vs. 17.6%, *p* < .001) were higher for patients with dementia. After standardizing for confounding variables, dementia remained an independent risk factor for death (HR 1.90; CI 1.37–2.65; *p* < .001) and MACE (HR 1.73; CI 1.30–2.31; *p* < .001), as well as in propensity score matched analysis (HR 1.54; CI: 1.03–2.28; *p* < .001 and HR 1.49; CI: 1.09–2.02; *p* < .001, respectively).

**Conclusions:**

Dementia is an independent predictor of worse outcomes in patients undergoing PCI for AMI. Future intervention and specialized healthcare measures to mitigate this risk is warranted.

## 1 INTRODUCTION

The rate of acute myocardial infarction (AMI) is a growing concern in the elderly population. The global prevalence of dementia more than doubled from 1990 to 2016 and is expected to increase from an estimated 57.4 million cases in 2019 to an estimated 152.8 million cases in 2050.^1^, ^2^ Additionally, the rates of ischemic heart disease (IHD) are expected to increase from 1655 to 1845 cases per 100 000 people in 2030.^3^ The elderly populations continues to grow as well, with predictions assuming that by 2050, 1 in 6 individuals will be over the age of 65, compared to 1 in 11 in 2019.^4^ As the elderly population increases, the incidence of IHD and AMI in this cohort will rise as well.^3^

In patients with acute myocardial infarction (AMI), dementia has an impact on whether patients receive percutaneous coronary intervention (PCI). Several studies have demonstrated that patients who have dementia are considerably less likely to receive PCI.^5^, ^6^, ^7^, ^8^ This has been hypothesized to be due to a myriad of factors, including the preference for a higher quality of life over an attempt to decrease mortality by invasive measures in this patient population. Another explanation might stem from the vulnerability to side effects or drug–drug interactions of patients suffering from dementia. However, after addressing and standardizing for these clinical measures, patients with dementia still received lower rates of interventional procedures.^7^, ^8^

Nevertheless, elderly patients, specifically octogenarians, have experienced similar benefits in ischemic complications when treated by PCI for AMI, when compared to younger patients.^9^ It is therefore becoming increasingly important to address outcomes in this specific growing population of patients with dementia who experience an AMI and are treated by PCI. In this study, based on a large registry of consecutive PCI patients, we examined the rates of 1‐year mortality and major adverse cardiac events (MACE) following the procedure in patients suffering from dementia and AMI.

## 2 METHODS

### 2.1 Study design

The present study is based on a prospectively collected PCI registry from the Rabin Medical Center in Petach Tikva—Israel, which includes 2 campuses—Beilinson and Hasharon hospitals. The registry includes consecutive patients treated with PCI from January 2004 through December 2020. The data is continuously entered into an ongoing registry for purposes of recording and monitoring patient‐related parameters, clinical events, and angiographic findings.

**Figure 1:**
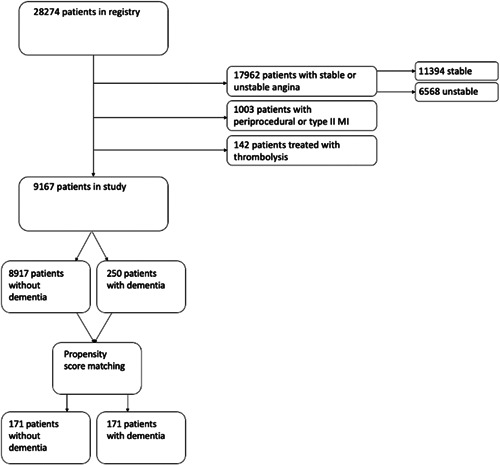
Patients included in study. Methodology for patient inclusion. Of the 28 274 patients who received PCI for AMI, only those who had STEMI or NSTEMI were included. Those with stable or unstable angina, or with periprocedural or type II MI were excluded. AMI, acute myocardial infarction; NSTEMI, non‐ST‐elevation myocardial infarction; PCI, percutaneous coronary intervention; STEMI, ST‐elevation myocardial infarction.

Of the 28 274 patients in the registry, we included only patients who were treated for ST‐elevation (STEMI) or non‐ST‐elevation myocardial infarction (NSTEMI) (Figure 1). Myocardial infarction was defined as detection of myocardial injury along with a clinical presentation of myocardial infarction, which may include symptoms such as chest or epigastric discomfort during exertion or rest.^10^ STEMI and NSTEMI differed from each other by extent of myocardial ischemia, evidenced by ECG findings. Patients were excluded if they presented with stable or unstable angina, if they were treated with thrombolysis instead of PCI (<1% of cases), in cases of periprocedural infarction or type‐2 myocardial infarction, or if they were ineligible for stent placement. We then collected 1‐year outcomes of patients with or without dementia. Dementia was defined by any of the following terms: “dementia” or “cognitive dysfunction” or “cognitive decline” or “cognitive impairment” or “Alzheimer's disease.”^11^ The study protocol and data collection was approved by the local Institutional Review Board.

### 2.2 Interventional procedure

All patients provided explicit written informed consent to undergo cardiac catheterization. Precatheterization treatment consisted of aspirin and unfractionated heparin (70 U/kg). Clopidogrel 300 or 600 mg, prasugrel 60 mg, or ticagrelor 180 mg was administered as a loading dose before or immediately after PCI. The utilization of glycoprotein IIb/IIIa inhibitors (GP2b3a) and choice of stent, as well as other therapeutic modalities such as mechanical thrombectomy and distal protection devices, were left to the discretion of the primary operator. All stents were implanted with moderate‐to‐high deployment pressure (12 to 16 atm). All patients received dual antiplatelet therapy with aspirin 100 mg daily and a thienopyridine (clopidogrel, prasugrel, or ticagrelor) for at least 12 months after PCI unless bleeding events caused premature cessation of dual antithrombotic treatment.

### 2.3 Endpoints

Immediate and in‐hospital events were prospectively collected in the institutional database. During follow‐up, patients completed standardized questionnaires for clinical events either by telephone (e.g., with the patient or with a family member) or in the outpatient clinics at 6‐month intervals. When indicated, records from peripheral hospitals were acquired to verify the events in the follow‐up period. All events were further confirmed and adjudicated by the institutional clinical events adjudication committee. Survival status at follow‐up was assessed by review of municipal civil registries at 1 year. Clinical outcomes included all‐cause mortality and MACE, which comprised death, MI, target vessel revascularization, and subsequent coronary artery bypass graft surgery. Renal failure was defined as glomerular filtration rate below 50 ml/min/1.73 m^2^ (according to the Modification of Diet in Renal Disease formula), anemia was defined as hemoglobin levels lower than 13.0 g/dl for men and 12.0 g/dl for women. Findings were compared between patients with dementia and those without.

### 2.4 Statistical analysis

Continuous data are summarized as mean and SD or median and interquartile range and were compared using Student *t*‐tests or analyses of variance. Categorical variables are presented as frequency and were compared by *χ* ^2^ or Fisher's exact tests. The normality of variable distributions was assessed using the Kolmogorov–Smirnov test. Time‐to‐event curves were constructed using the Kaplan–Meier method and compared using log‐rank test. Cox regression analyses were performed to identify independent predictors of the primary endpoint. Covariates for the Cox model were chosen according to their known association with dementia and outcomes, and included age, sex, diabetes mellitus, renal failure, peripheral artery disease, left ventricular ejection fraction (for each 1% increase), previous oncological disease, ST‐elevation myocardial infarction, trans radial access and dementia. Finally, due to several differences in baseline characteristics, we compiled a cohort of propensity score matched patients with a 1:1 ratio between patients with dementia and controls. The propensity score was derived from a multivariate logistic regression model that included dementia, considered as the independent (outcome) variable, and all baseline clinical characteristics and procedural characteristics as covariates. The propensity score matched cohort was analyzed for the main combined outcome. Effect sizes are presented as odds ratios and 95% confidence intervals. All statistical analyses were performed with IBM SPSS statistics V.28 software. A *p* < .05 was considered statistically significant.

## 3 RESULTS

Our study consisted of 9167 patients, of which 8917 did not suffer from dementia and 250 did. Mean age was 77.4 ± 9.4 and 63.6 ± 12.7 for patients with and without dementia, respectively (*p* < .001). 32.4% of the patients with dementia were female compared to 24.2% for those without (*p* = .003). Other baseline characteristics, including the prevalence of previous coronary artery bypass graft, history of atrial fibrillation and prior peripheral vascular disease—did not differ between the groups (Table 1).

**Table 1:** Baseline characteristics

Parameter	Control (*n* = 8917)	Dementia (*n* = 250)	*p* Value
Age	63.6 ± 12.7	77.4 ± 9.4	<.001
Female sex (%)	24.2	32.4	.003
Diabetes mellitus (%)	42.4	54.0	<.001
Hypertension (%)	69.2	84.0	<.001
Prior smoking (%)	46.2	17.2	<.001
Prior CHF (%)	26.4	44.4	<.001
Prior COPD (%)	8.2	16.8	<.001
Prior PVD (%)	5.6	4.8	.569
Atrial fibrillation (%)	7.2	8.4	.452
Stroke (%)	6.7	24.8	<.001
Prior malignancy (%)	10.0	20.8	<.001
CABG (%)	8.2	10.8	.141
CHA_2_DS_2_VASc	3.4	5.3	<.001
CKD (%)	19.3	44.4	<.001

Abbreviations: CABG, coronary artery bypass graft; CHF, congestive heart failure; CKD, chronic kidney disease; COPD, chronic obstructive pulmonary disease; PVD, peripheral vascular disease.

Procedural characteristics are shown in Table 2. Importantly, there was no difference in rates of STEMI as opposed to NSTEMI between the two groups (*p* = .822). Patients with dementia were more likely to present in state of cardiogenic shock (4.8 vs. 2.5%, *p* = .026) and have a greater number of vessels involved (2.4 ± 0.7 vs. 2.2 ± 0.8, *p* ≤ .001) as part of the acute presentation of AMI.

**Table 2:** Procedural characteristics

Parameter	Control (*n* = 8917)	Dementia (*n* = 250)	*p* Value
EF (%)	52.8	49.6	.001
Unprotected LMCA (%)	3.0	7.6	<.001
Shock (%)	2.5	4.8	.026
No. of vessels involved	2.2 ± 0.8	2.4 ± 0.7	<.001
No. of territories	1.6 ± 0.8	1.7 ± 0.9	.043
STEMI (%)	31.9	31.2	.822
Radial approach (%)	46.1	33.2	<.001
Drug‐eluting stent (%)	90.9	91.0	.215
Symptoms to admission (hours)	3.81 ± 1.48	4.00 ± 1.51	.022
Admission to PCI, STEMI patients (hours)	0.92 ± 0.39	1.02 ± 0.42	.095
Hemoglobin A1C (%)	7.0 ± 1.8	7.2 ± 2.0	.443
Hemoglobin (g/dl)	13.6 ± 1.9	12.4 ± 2.0	<.001
Platelet count (×10^3^ mm^3^)	244.1 ± 79.5	249.7 ± 85.4	.277
Total cholesterol (mg/dl)	173.4 ± 46.1	158.9 ± 42.0	<.001
HDL (mg/dl)	40.3 ± 11.8	43.0 ± 12.8	.004
LDL (mg/dl)	102.5 ± 39.2	89.7 ± 33.0	<.001

Abbreviations: EF, ejection fraction; HDL, high‐density lipoprotein; LDL, low‐density lipoprotein; LMCA, left main coronary artery; STEMI, ST‐elevation myocardial infarct.

**Figure 2:**
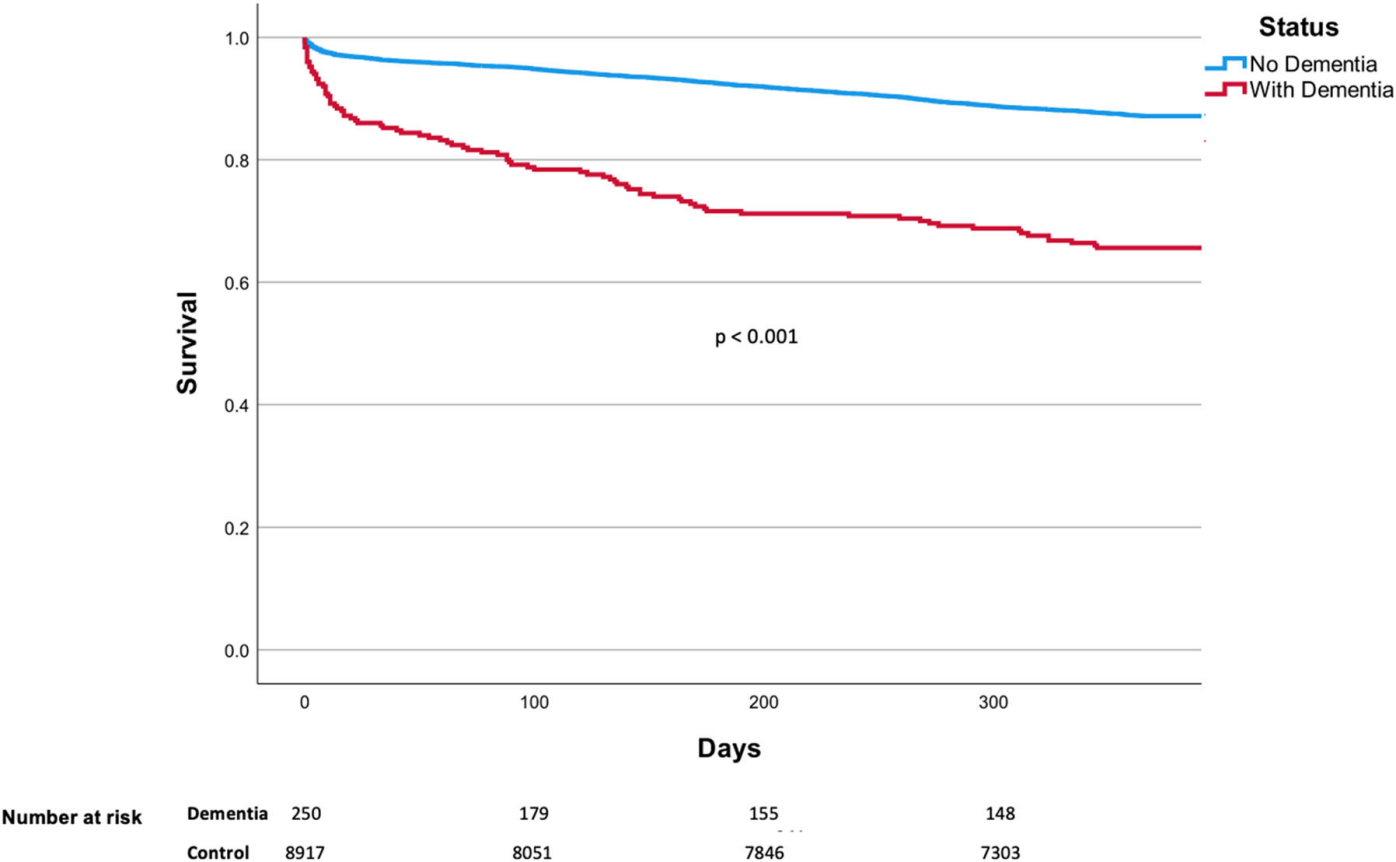
Kaplan–Meier Curves—Risk of death. Unadjusted rates of death dependent on patient's dementia status. In a 1‐year follow‐up period post‐PCI, patients with dementia had a 25.5% chance of death, compared to 9.8% for those without dementia.

**Figure 3:**
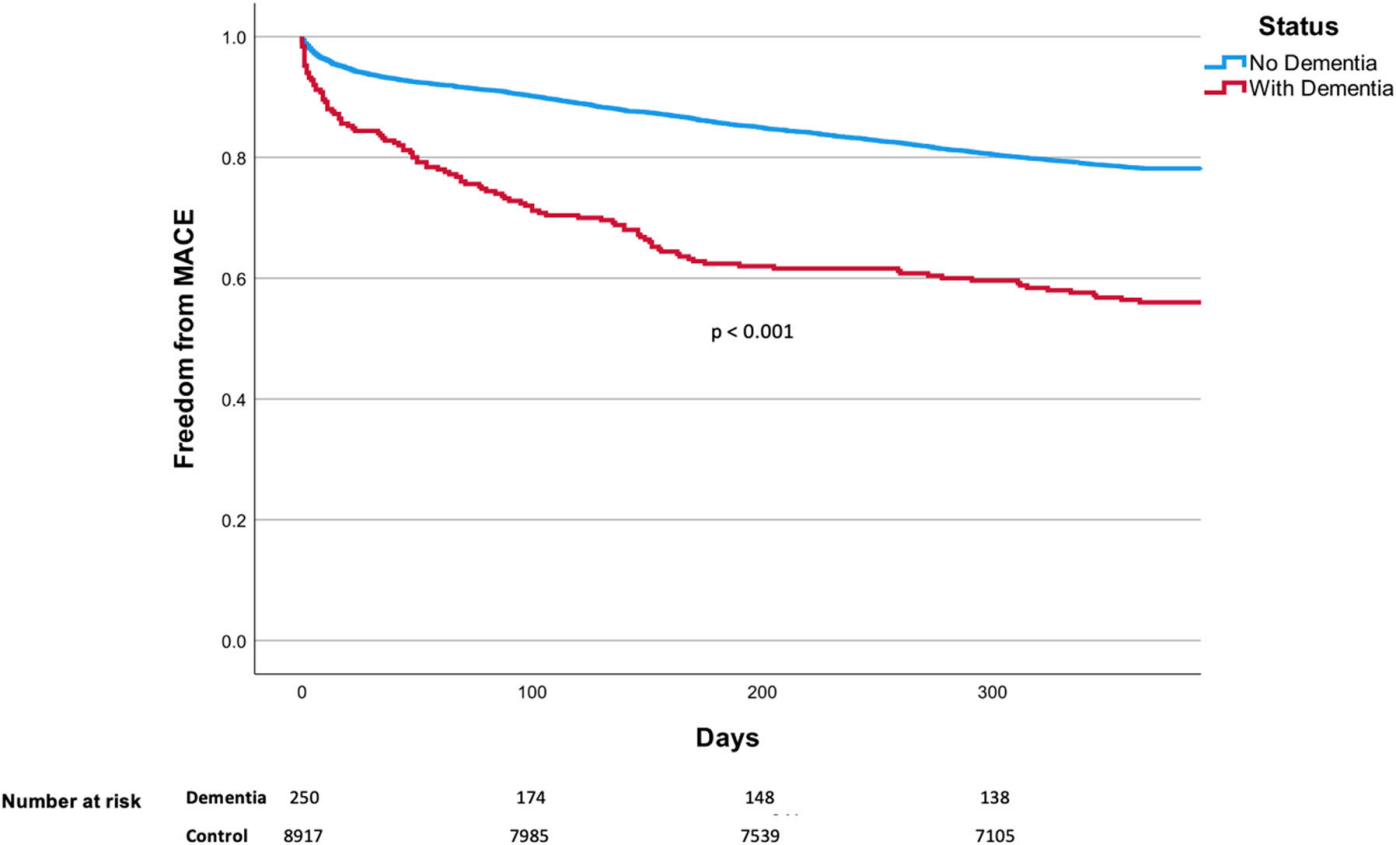
Kaplan–Meier Curves—Risk of MACE. Unadjusted rates of MACE dependent on patient's dementia status. In a 1‐year follow‐up period post‐PCI, patients with dementia had a 33.8% chance to encounter an event of MACE, compared to 17.6% for those without dementia. MACE, major adverse cardiac events; PCI, percutaneous coronary intervention.

The unadjusted cumulative probability of reaching the endpoint of death for patients with dementia at a 1‐year follow‐up period was 25.5 versus 9.8% for the control (*p* < .001). Comparatively, the MACE endpoint was reached 33.8 versus 17.6% for the control (*p* < .001). Kaplan–Meier curves demonstrating these unadjusted risks are shown in in Figures 2 and 3. Following Cox regression analysis, adjusting for differences in baseline characteristics, patients with dementia were 1.73 (95% CI 1.30–2.31; *p* < .001) and 1.90 (95% CI 1.37–2.65; *p* < .001) times more likely to suffer MACE and death, respectively, 1‐year after PCI (Supporting Information: Tables 1 and 2, Figures 1 and 2).

The propensity match score was able to form 171 matched pairs of patients with dementia and control patients, showing similar results. After propensity matched score analysis, mean age was 76.6 ± 9.2 and 76.4 ± 9.3 for patients with and without dementia, respectively. The cohort of patients with dementia were 32.3% female, 53.9% had diabetes, and 83.7% had hypertension. Those without dementia were 32.1% female, 53.8% had diabetes, and 83.5% had hypertension. Following Cox regression, patients presenting with dementia demonstrated higher rates of MACE (HR 1.49; CI: 1.09–2.02; *p* < .001) and death (HR 1.54; CI: 1.03–2.28; *p* < .001) than patients without dementia. Additional remaining baseline characteristics postpropensity matching as well as the KM curves demonstrating these results are included in Supporting Information: Table 3, Figures 3 and 4.

## 4 DISCUSSION

The current study demonstrates a significant independent association in the rate of adverse outcomes for patients who have dementia following PCI for AMI. The study shows that patients with dementia are more than two times more likely to suffer death and nearly two times more likely to encounter MACE 1‐year after their PCI. Also, after correcting for confounders, dementia remains an independent risk for adverse events.

The incidence of IHD and dementia is closely tied. Those with a history of IHD are, on average, at a 45% increased risk of developing cognitive impairment.^12^ The direct effect of large artery atherosclerosis is one of the mechanisms responsible for this shared correspondence, as it is a significant component for the development of vascular dementia. In addition to this direct influence, IHD has been shown to be correlated with increased senile plaque formation and reduced hippocampal size.^13^, ^14^, ^15^ Apart from the aforementioned correlations between IHD and dementia, both diseases share common risk factors, including obesity, type II diabetes mellitus, and hypercholesterolemia, among others.^12^, ^13^, ^16^ Nevertheless, when adjusting for these various cofounders, the association between cardiovascular disease and cognitive decline is still prominent.^17^ Several studies have examined the association between these two pathologies, but very few have examined the implications of cognitive impairment on the prognosis of patients with IHD who undergo PCI. While not equivalent to dementia, frailty is a marker that has been used to explore this topic.

In a study that assessed patients undergoing PCI for frail patients with stable angina or acute coronary syndrome, both mortality and length of hospital stay increased: 30‐day mortality was 4.9% versus 1.1% for nonfrail patients (*p* = .01) and length of hospital stay was 2.9 ± 5.6 versus 1.7 ± 3.1 days for nonfrail patients (*p* < .001).^18^ Similarly, in a cohort of 62 patients who presented with STEMI, and after adjustment for common confounders, including BMI and troponin levels, higher frailty scores were associated with increased in‐hospital mortality and failure of discharge to home. Higher frailty scores were 6.28 times more likely to suffer in‐hospital mortality and 16.69 times more likely to not be discharged home.^19^ In these studies, frailty was defined using the Canadian Study of Health and Aging—Clinical Frailty Score, which has been demonstrated to be correlated with cognitive impairment.^20^ Hamonangan and colleagues defined frailty using the frailty phenotype criteria, which has also been demonstrated to be highly associated with cognitive status.^21^ However, in this study that only looked at MACE for elderly patients who had undergone PCI for coronary artery disease, no significant association was found for frail patients who suffered MACE after PCI: 8.19% of cases versus 5.12% of cases for nonfrail patients (*p* > .05).^22^

While a patient's frailty status is not equivocal to a patient's clinical dementia status, higher mortality rates, worse long‐term prognoses, and longer hospital stays were all associated with higher frailty scores.^18^, ^23^, ^24^ Most of the studies investigating the association between higher levels of clinical frailty and worse outcomes for patients with acute coronary syndrome or CAD have found it to be significant.^18^, ^19^, ^23^, ^24^, ^25^ However, it is also important to study the independent effects of cognitive dysfunction on outcomes in this cohort. To that extent, this study is the largest and first of its kind to look at the independent effects of dementia on outcomes for patients with STEMI or NSTEMI who undergo PCI.

Other independent associations that have been demonstrated to lead to higher‐all cause mortality in patients with AMI include older age,^26^ lower hemoglobin levels,^27^ and progressive kidney dysfunction.^28^ Even in cases of therapy with PCI, complication rate is highly correlated with age. Older patients tend to have higher rates of in‐hospital death, restenosis, and postprocedural complications, as compared to younger patients.^29^, ^30^ Nevertheless, PCI remains the preferred treatment over medical therapy for this aging population suffering from AMI.^29^, ^31^ Similarly, lower levels of hemoglobin and progressive kidney dysfunction presume a worse prognosis. Our data demonstrated that the cohort of patients with dementia did have statistically significant differences in age, hemoglobin levels, and rates of chronic kidney disease. However, even after adjusting for these major factors, patients with dementia were 1.7 times more likely to suffer from MACE and 1.9 times more likely to die at the 1‐year interval.

An explanation for the increase in MACE and death for this elderly population could stem from a delay from initial symptoms to admission in the hospital. In our analysis, patients with dementia were admitted 4.00 ± 1.51 h after initial symptoms compared to 3.81 ± 1.48 h for patients without dementia (*p* < .05). Patients who suffer from dementia may not be as aware of their symptoms and requirement for medical attention as their controls. This decreased awareness may lead to delays in hospital arrivals and increase in the percentage of ischemic burden upon presentation. Even with PCI performed, the prognosis in this situation would be less favorable. In our study, there were no differences in door‐to‐balloon times in this combined STEMI/NSTEMI cohort, but there was a significant difference in patient delay before admission. Additionally, patients with cognitive impairment are also more likely to suffer from other comorbidities. The worse overall health that these patients initially present with may confer a worse overall outcome, even after PCI.

With a growing population of elderly patients and patients with dementia, along with a projected increase in cases of IHD, consideration should be placed on how to improve the prognosis for this population of patients with dementia suffering from AMI. Appropriate surveillance of this population will likely lead to earlier assessment and revascularization for patients with IHD. To increase awareness of symptoms and avoid delays in hospitalization, frequent screening via direct patient visits, as well as telemedicine, can be used. Implementing ECG transmission systems from home has been show to improve triage at the hospital and lead to decreased delays in treatment.^32^ When medications and frequent surveillance are not enough to prevent the progression of IHD, the usage of PCI should be more effectively assessed. Compared to younger patients, elderly patients have a 2‐to‐4‐fold increase in the risk of morbidity and mortality.^33^ When inappropriately used, PCI can lead to complications such as stent thrombosis and restenosis.^34^ The development and increased usage of fractional flow reserve instead of anatomic angiography in the assessment of PCI usage in nonculprit AMI lesions may lead to a better prognosis for this cohort.

## 5 LIMITATIONS

First, this is an observational study, meaning the generalizability of its findings are limited. This also includes the definition of dementia, derived from ICD‐9 and ICD‐10 diagnoses in the patient history. Therefore, correlations can be used on the population observed, but causality cannot be concluded. Second, there was a relatively small number of patients with dementia, which again limits the generalizability of such a study. Third, the diagnosis of dementia in our study is clinical. We do not have objective neuropsychological testing documentation to assess severity. Although it is likely that most patients with the diagnosis have cognitive impairment, it is not confirmed. Finally, our study was absent of a clinical frailty index. Past studies have used some variation of clinical frailty to demonstrate the association between PCI outcomes and frailty. The lack of an index limits the generalizability of our study. Nevertheless, our use of dementia as an associative factor provides a novel perspective on the outcomes for PCI in this elderly cohort and is the largest thus far to report of outcomes in this important and gradually growing patient population suffering from AMI.

## 6 CONCLUSION

Our study demonstrates worse outcomes for patients with dementia treated by PCI for AMI than those without dementia. These findings provide clinicians with a better understanding of the prognosis of patients with dementia and emphasize the need for novel methods of specialized healthcare delivery to mitigate this added risk.

## CONFLICT OF INTEREST

The authors declare no conflict of interest.

## Supporting information

Supplementary information.

Supplementary information.

Supplementary information.

Supplementary information.

Supplementary information.

Supplementary information.

Supplementary information.

Supplementary information.

## Data Availability

Original research data will be available upon request.
