# Supplementary figures and images for: The independent impact of dementia in patients undergoing percutaneous coronary intervention for acute myocardial infarction
Source: Clin Cardiol. 2023 Jan 12;46(3):279–86. doi: 10.1002/clc.23967 (PMC10018096; doi:10.1002/clc.23967)

Supplementary Fig. 1

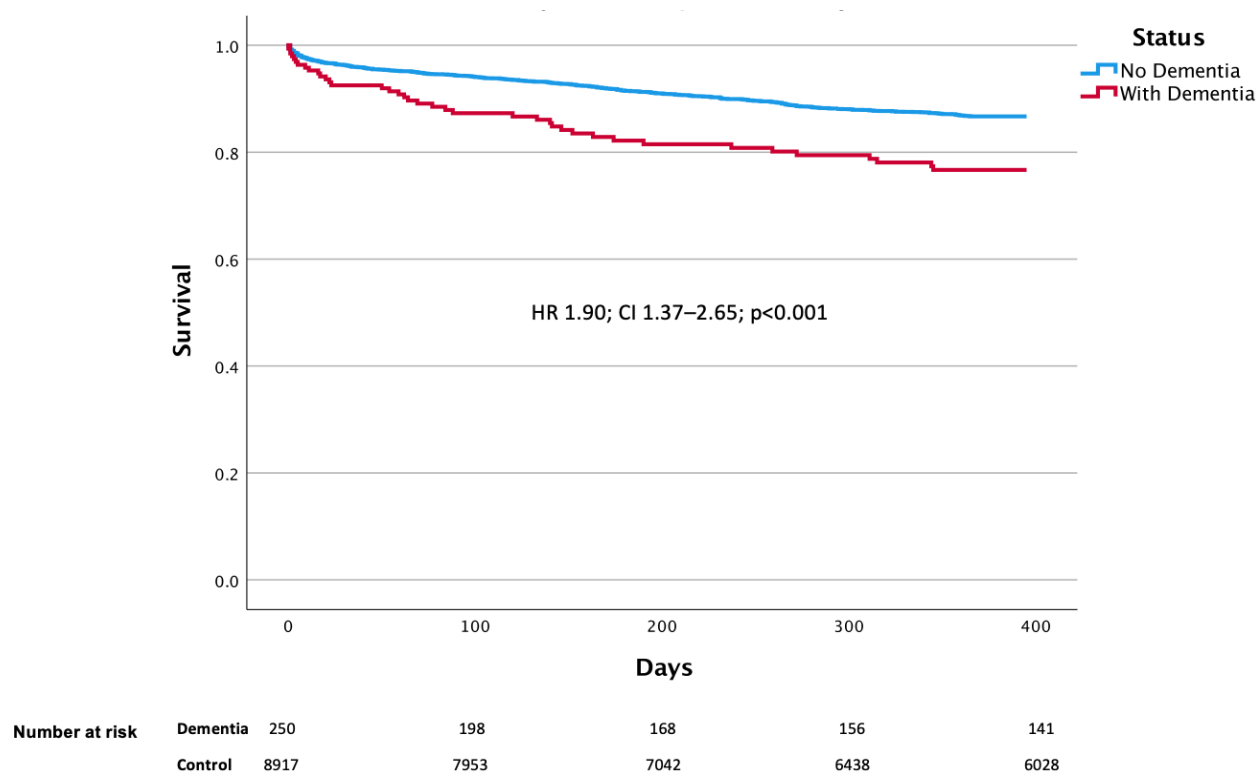

Supplement: Supplementary file 1 — Supplementary information. [file CLC-46-279-s001.pdf]

Supplementary Fig. 2

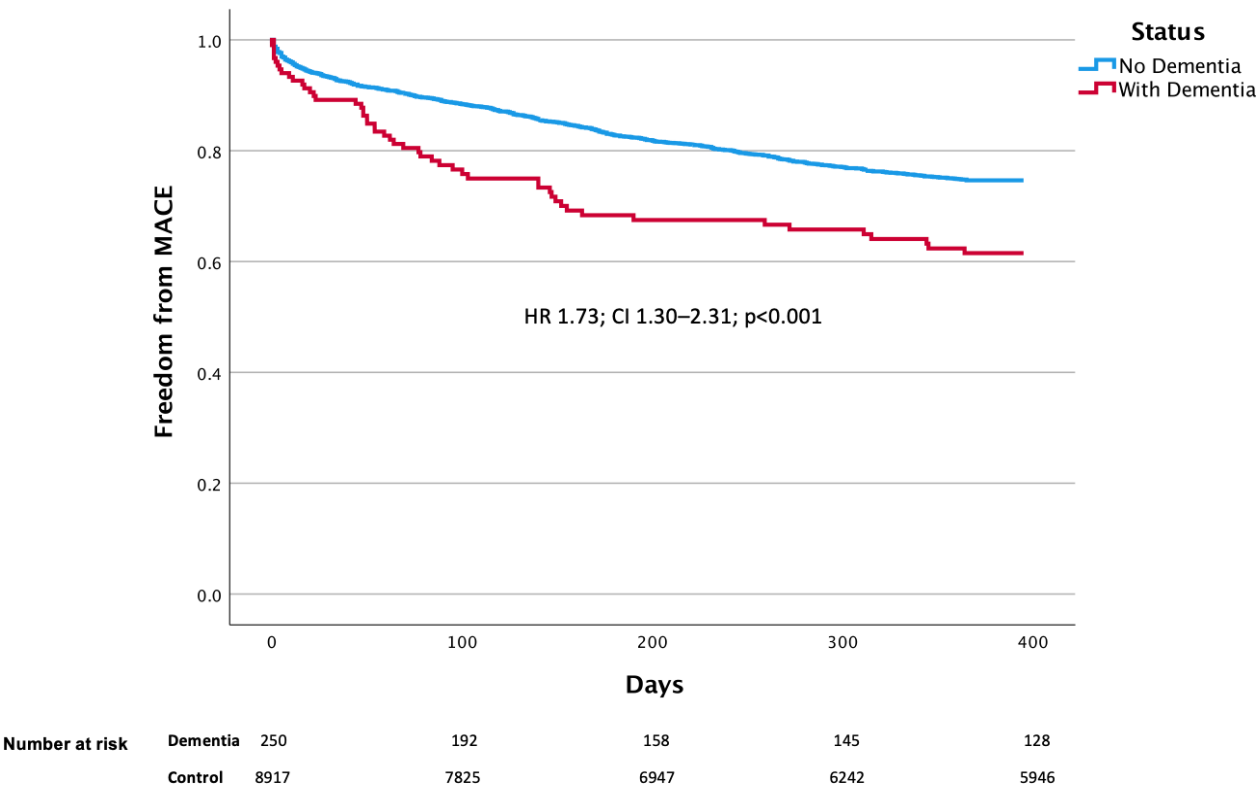

Supplement: Supplementary file 2 — Supplementary information. [file CLC-46-279-s008.pdf]

Supplementary Fig. 3

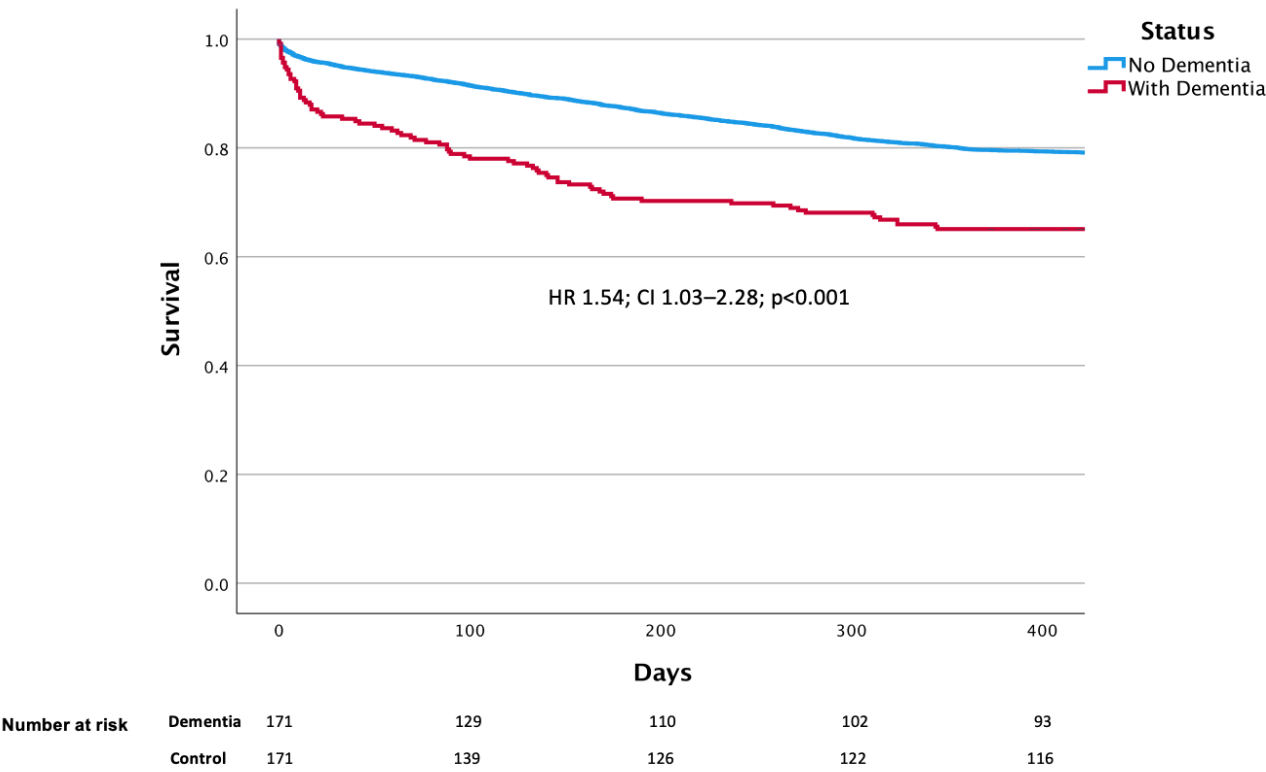

Supplement: Supplementary file 3 — Supplementary information. [file CLC-46-279-s004.pdf]

Supplementary Fig. 4

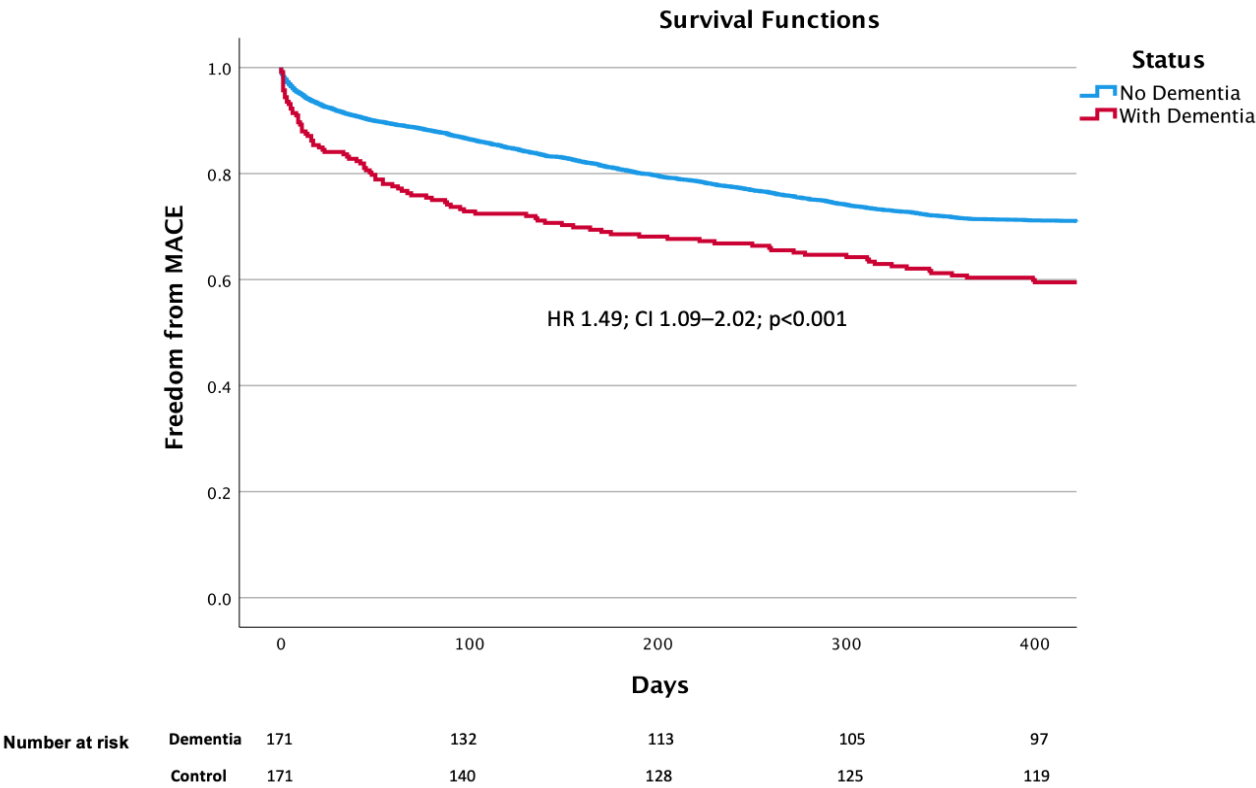

Supplement: Supplementary file 4 — Supplementary information. [file CLC-46-279-s002.pdf]
