# Supplementary material for: The independent impact of dementia in patients undergoing percutaneous coronary intervention for acute myocardial infarction
Source: Clin Cardiol. 2023 Jan 12;46(3):279–86. doi: 10.1002/clc.23967 (PMC10018096; doi:10.1002/clc.23967)
Supplement: Supplementary file 5 — Supplementary information. [file CLC-46-279-s003.docx]

**Supplementary Fig. 1 Kaplan Meier Curves - Adjusted risk of death**

Multivariate regression analysis, adjusted for by common confounding variables. After adjustment and in a 1-year follow up period post-PCI, patients with dementia were 1.9 times more likely to die

**Supplementary Fig. 2 Kaplan Meier Curves- Adjusted risk of MACE**

Multivariate regression analysis, adjusted for by common confounding variables. After adjustment and in a 1-year follow up period post-PCI, patients with dementia were 1.73 times more likely to encounter an event of MACE

**Supplementary Fig. 3** **Propensity Matched Score Kaplan Meier Curve – Adjusted risk of death**

Matching for baseline characteristics, in a propensity matched analysis, patients with dementia were more likely to suffer death in a 1 year-follow up compared to their non-dementia control group (HR 1.54, CI 1.03-2.28; p < 0.001).

**Supplementary Fig. 4** **Propensity Matched Score Kaplan Meier Curve – Adjusted risk of MACE**

Matching for baseline characteristics, in a propensity matched analysis, patients with dementia were more likely to suffer MACE in a 1 year-follow up compared to their non-dementia control group (HR 1.49, CI 1.09-2.02; p < 0.001).
