# Supplementary material for: The independent impact of dementia in patients undergoing percutaneous coronary intervention for acute myocardial infarction
Source: Clin Cardiol. 2023 Jan 12;46(3):279–86. doi: 10.1002/clc.23967 (PMC10018096; doi:10.1002/clc.23967)
Supplement: Supplementary file 6 — Supplementary information. [file CLC-46-279-s006.docx]

Supplementary Table 1: Adjusted Parameters for Cox Regression on 1-year MACE

| **Parameter** | **HR Hazard Ratio** | **95% CI** | **P-value** |
| --- | --- | --- | --- |
| CKD | 1.91 | 1.64 - 2.22 | <0.001 |
| Age over 65 | 1.21 | 1.03 – 1.42 | 0.022 |
| Anemia | 1.09 | 0.93 – 1.27 | 0.298 |
| Radial approach | 0.81 | 0.70 – 0.94 | 0.004 |
| STEMI | 1.11 | 0.74 – 1.65 | 0.618 |
| Symptoms to admission (hours) | 1.20 | 0.81-2.04 | 0.742 |
| LVEF | 0.99 | 0.98- 1.00 | 0.005 |
| Prior PVD | 1.49 | 1.21 – 1.82 | <0.001 |
| Female sex | 0.89 | 0.75 – 1.04 | 0.135 |
| Diabetes mellitus | 1.04 | 0.90 – 1.20 | 0.606 |
| Prior malignancy | 1.44 | 1.21 – 1.71 | <0.001 |
| Prior dementia | 1.73 | 1.30 – 2.31 | <0.001 |

CKD = chronic kidney disease; LVEF = left ventricular ejection fraction; PVD = peripheral vascular disease.
