# Supplementary material for: The independent impact of dementia in patients undergoing percutaneous coronary intervention for acute myocardial infarction
Source: Clin Cardiol. 2023 Jan 12;46(3):279–86. doi: 10.1002/clc.23967 (PMC10018096; doi:10.1002/clc.23967)
Supplement: Supplementary file 7 — Supplementary information. [file CLC-46-279-s005.docx]

Supplementary Table 2: Adjusted Parameters for Cox Regression on 1-year Death

| **Parameter** | **HR Hazard Ratio** | **95% CI** | **P-value** |
| --- | --- | --- | --- |
| CKD | 2.24 | 1.84 – 2.74 | <0.001 |
| Age over 65 | 1.68 | 1.34 – 2.12 | <0.001 |
| Anemia | 1.13 | 0.92 – 1.38 | 0.260 |
| Radial approach | 0.86 | 0.71 – 1.04 | 0.112 |
| STEMI | 1.23 | 0.74 – 2.05 | 0.419 |
| Symptoms to admission (hours) | 1.09 | 0.69-2.27 | 0.386 |
| LVEF | 0.98 | 0.97 – 0.99 | <0.001 |
| Prior PVD | 1.37 | 1.05 – 1.79 | 0.020 |
| Female sex | 0.97 | 0.79 – 1.19 | 0.748 |
| Diabetes mellitus | 0.83 | 0.69 – 1.01 | 0.056 |
| Prior malignancy | 1.62 | 1.31 – 2.00 | <0.001 |
| Prior dementia | 1.90 | 1.37 – 2.65 | <0.001 |

CKD = chronic kidney disease; LVEF = left ventricular ejection fraction; PVD = peripheral vascular disease
