# Supplementary material for: The independent impact of dementia in patients undergoing percutaneous coronary intervention for acute myocardial infarction
Source: Clin Cardiol. 2023 Jan 12;46(3):279–86. doi: 10.1002/clc.23967 (PMC10018096; doi:10.1002/clc.23967)
Supplement: Supplementary file 8 — Supplementary information. [file CLC-46-279-s007.docx]

Supplementary Table 3: Propensity Matched Scoring Analysis Baseline Characteristics

| **Parameter** | **Control (n = 171)** | **Dementia (n = 171)** |
| --- | --- | --- |
| Age | 76.4±9.3 | 76.6±9.2 |
| Female sex (%) | 32.1 | 32.3 |
| Diabetes mellitus (%) | 53.8 | 53.9 |
| Hypertension (%) | 83.5 | 83.7 |
| Prior smoking (%) | 18.2 | 18.1 |
| Prior CHF (%) | 43.8 | 44.1 |
| Atrial fibrillation (%) | 7.9 | 8.0 |
| Stroke (%) | 23.1 | 23.9 |
| Prior malignancy (%) | 19.7 | 20.1 |
| CABG (%) | 10.5 | 10.5 |
| CKD (%) | 43.2 | 43.6 |
| EF (%) | 50.1 | 49.9 |
| Unprotected LMCA (%) | 7.1 | 7.4 |
| Shock (%) | 4.7 | 4.7 |
| STEMI (%) | 31.6 | 31.4 |
| Radial approach (%) | 38.4 | 37.8 |
| Drug-eluting stent (%) | 90.9 | 91.0 |
| Symptoms to admission (hours) | 3.9±1.5 | 3.9±1.5 |
| Hemoglobin (g/dl) | 12.9±2.0 | 12.6±1.9 |
| LDL (mg/dl) | 90.8±39.0 | 89.9±37.2 |

CABG = coronary artery bypass graft; CHF = congestive heart failure; CKD = chronic kidney disease; LMCA = left main coronary artery; STEMI = ST-elevation myocardial infarct

* All p value comparisons are non-significant.
